# Supplementary material for: Endothelial Unc5B controls blood-brain barrier integrity
Source: Nat Commun. 2022 Mar 4;13:1169. doi: 10.1038/s41467-022-28785-9 (PMC8897508; doi:10.1038/s41467-022-28785-9)
Supplement: Supplementary file 1 — Supplementary Information [file 41467_2022_28785_MOESM1_ESM.pdf]

Supp Fig. 1

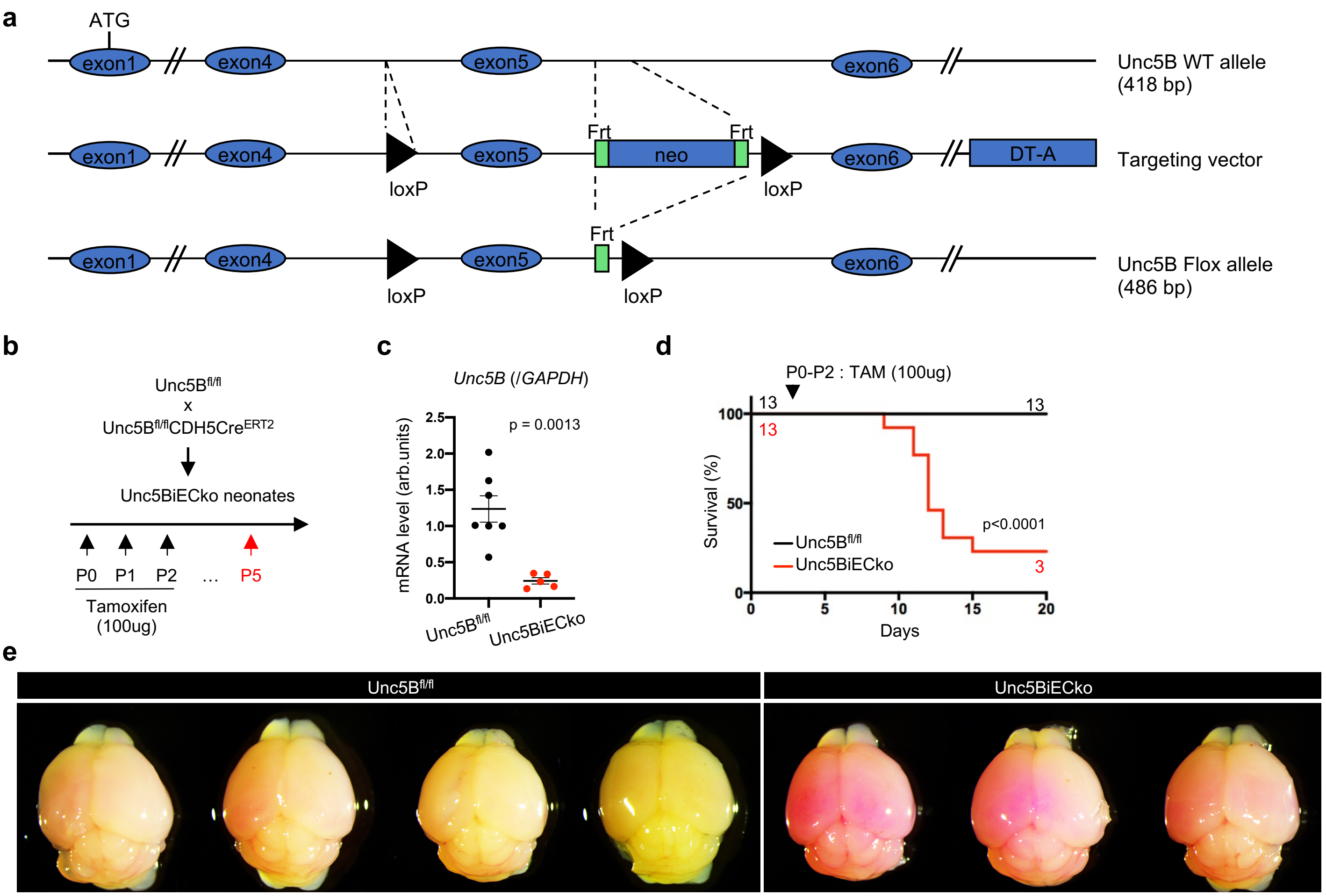
a
ATG
exon1
exon4
exon5
exon6
Unc5B WT allele
(418 bp)
loxP
Frt
neo
DT-A
Targeting vector
Unc5B Flox allele
(486 bp)
b
Unc5B fl/fl
x
Unc5B fl/fl CDH5Cre ERT2
Unc5BiECko neonates
P0
P1
P2
…
P5
Tamoxifen
(100ug)
c
Unc5B (/GAPDH)
mRNA level (arb.units)
p = 0.0013
Unc5B fl/fl
Unc5BiECko
d
P0-P2 : TAM (100ug)
Survival (%)
13
13
13
3
p<0.0001
Unc5B fl/fl
Unc5BiECko
Days
e
Unc5B fl/fl
Unc5BiECko

Supplemental Figure 1:

(a) Diagram illustrating generation of the *Unc5B* Flox allele. (b) *Unc5B* gene deletion strategy using tamoxifen injection in postnatal mice. (c) qPCR analysis of *Unc5B* mRNA level on isolated P5 mouse lung endothelial cells, n=7 *Unc5B*$^{fl/fl}$ and n=5 *Unc5BiECko* brains. Each dot represents one mouse. One control mouse was set as 1. (d) Survival curve after neonatal *Unc5B* gene deletion, n=13 *Unc5B*$^{fl/fl}$ and n=13 *Unc5BiECko* (exact p-value = 0.000055). (e) Cadaverine leakage in P5 brains 2h after intraperitoneal cadaverine injection. All data are shown as mean+/-SEM. Two-sided Mann-Whitney U test was performed for statistical analysis between two groups. Mantel-cox test was performed for statistical analysis of the survival curve. Source data are provided as a Source Data file.

Supp Fig. 2

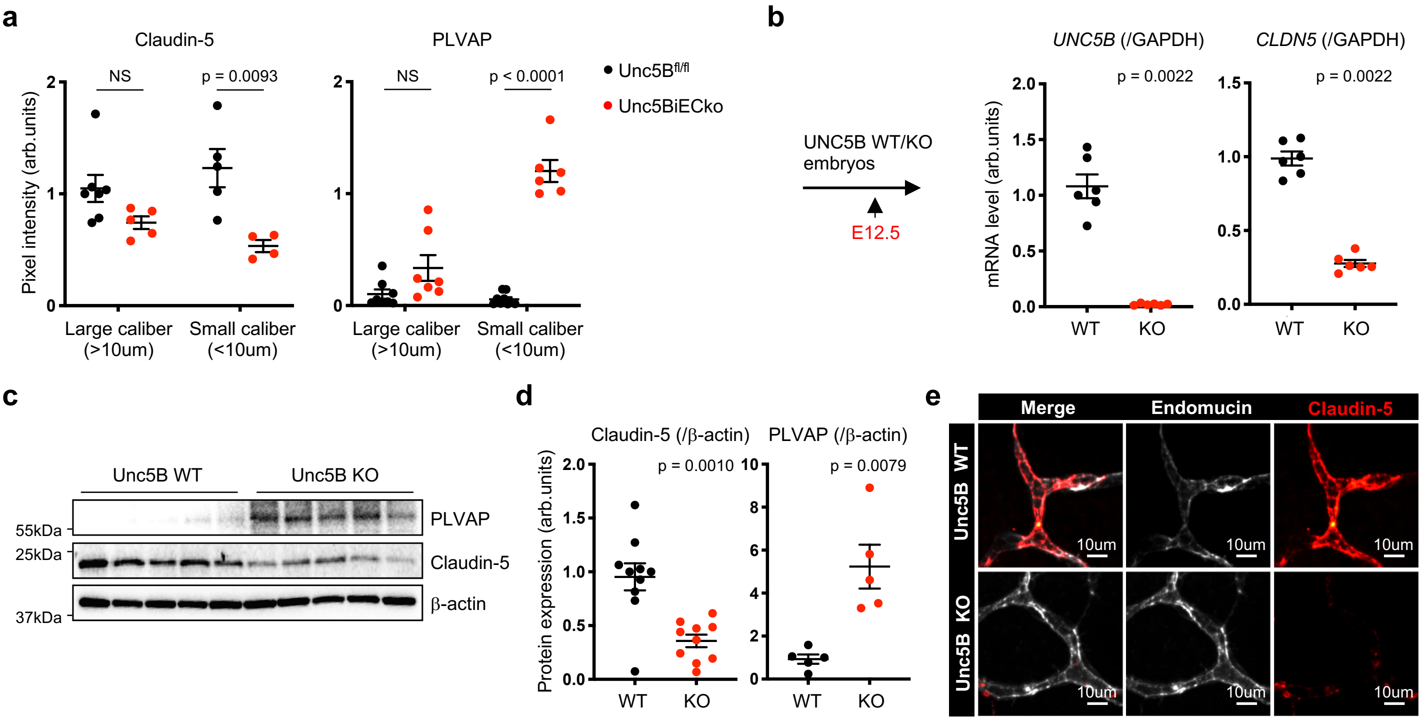

a
Claudin-5
PLVAP
NS
p = 0.0093
NS
p < 0.0001
Unc5Bfl/fl
Unc5BiECko
Pixel intensity (arb.units)
Large caliber (>10um)
Small caliber (<10um)
b
UNC5B WT/KO embryos
E12.5
UNC5B (/GAPDH)
CLDN5 (/GAPDH)
p = 0.0022
p = 0.0022
mRNA level (arb.units)
WT
KO
c
Unc5B WT
Unc5B KO
55kDa
25kDa
37kDa
PLVAP
Claudin-5
β-actin
d
Claudin-5 (/β-actin)
PLVAP (/β-actin)
p = 0.0010
p = 0.0079
Protein expression (arb.units)
e
Merge
Endomucin
Claudin-5
Unc5B WT
Unc5B KO
10um

Supplemental Figure 2:

(a) Quantification of Claudin-5 and PLVAP immunostaining on brain sections in larger vessels (diameter > 10um) versus smaller vessel (diameter < 10um). Each dot represents the mean of several images, n=5/7 *Unc5B*$^{fl/fl}$ and n=4/5 *Unc5BiECko* for quantification of Claudin-5 expression, n=8/9 *Unc5B*$^{fl/fl}$ and n=6/7 *Unc5BiECko* for quantification of PLVAP expression (exact p-value for small caliber vessel = 0.00000000009). One control mouse was set as 1. (b) qPCR analysis on E12.5 brain mRNA extracts, n=6 *Unc5B WT* and n=6 *Unc5B KO* embryos. Each dot represents one embryo. One control embryo was set as 1. (c,d) Western blot (c) and quantification (d) of E12.5 brain protein extracts. n=10 *Unc5B WT* and n=10 *Unc5B KO* embryos for Claudin-5 protein quantification, n=5 *Unc5B WT* and n=5 *Unc5B KO* embryos for PLVAP protein quantification. Each dot represents one embryo. One control embryo was set as 1. (e) Whole-mount hindbrain immunofluorescence staining with the indicated antibodies and confocal imaging of E12.5 embryos, reproduced on n=5 *Unc5B WT* and n=5 *Unc5B KO* embryos. All data are shown as mean+/-SEM. NS: non-significant, two-sided Mann-Whitney U test was performed for statistical analysis. Source data are provided as a Source Data file.

Supp Fig. 3

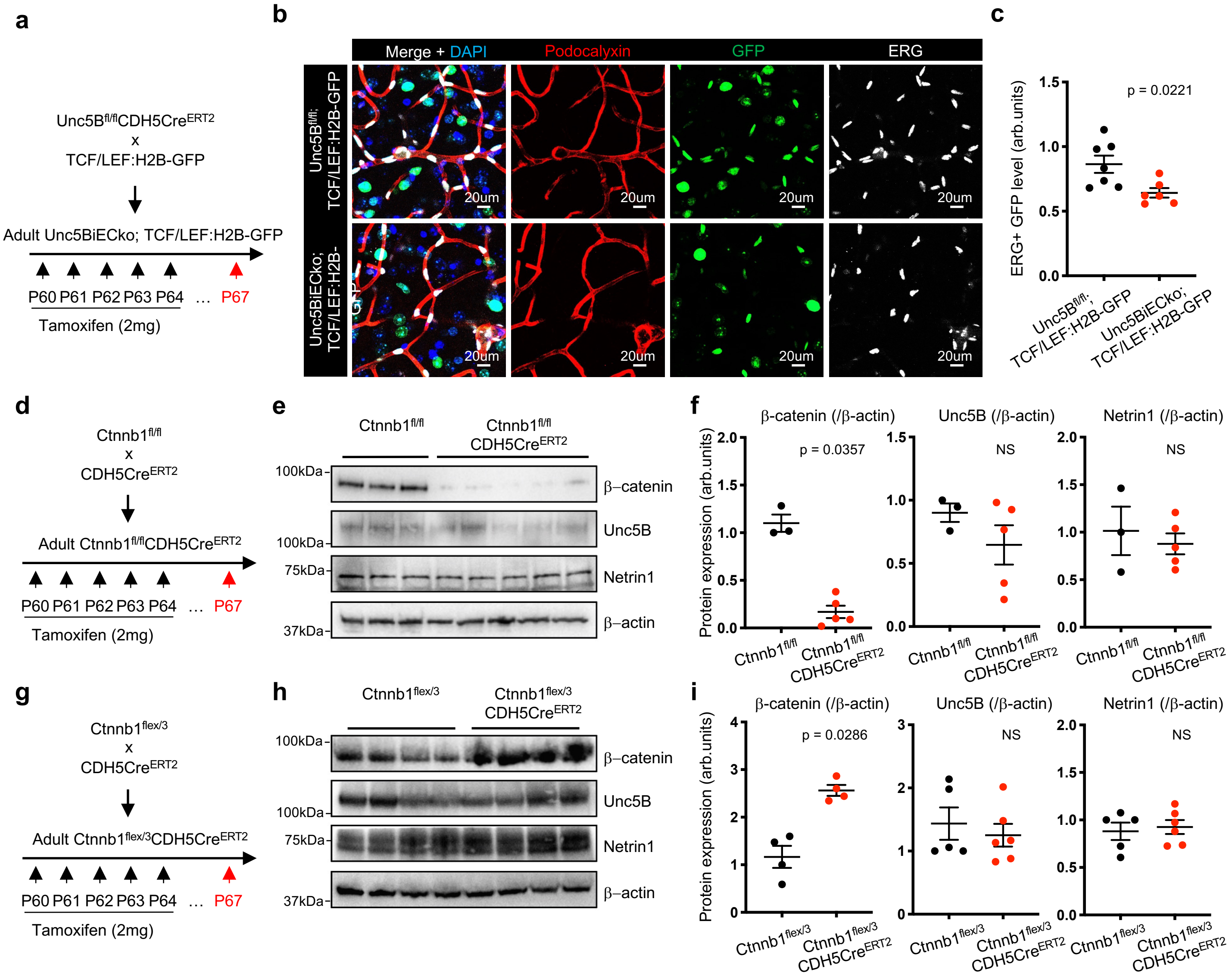

a
Unc5Bfl/flCDH5CreERT2
x
TCF/LEF:H2B-GFP
Adult Unc5BiECko; TCF/LEF:H2B-GFP
P60 P61 P62 P63 P64 … P67
Tamoxifen (2mg)
b
Merge + DAPI
Podocalyxin
GFP
ERG
Unc5Bfl/fl; TCF/LEF:H2B-GFP
Unc5BiECko; TCF/LEF:H2B-GFP
20um
c
p = 0.0221
ERG+ GFP level (arb.units)
Unc5Bfl/fl; TCF/LEF:H2B-GFP
Unc5BiECko; TCF/LEF:H2B-GFP
d
Ctnnb1fl/fl
x
CDH5CreERT2
Adult Ctnnb1fl/flCDH5CreERT2
P60 P61 P62 P63 P64 … P67
Tamoxifen (2mg)
e
Ctnnb1fl/fl
Ctnnb1fl/fl CDH5CreERT2
100kDa
75kDa
37kDa
β–catenin
Unc5B
Netrin1
β–actin
f
β-catenin (/β-actin)
Unc5B (/β-actin)
Netrin1 (/β-actin)
Protein expression (arb.units)
p = 0.0357
NS
NS
Ctnnb1fl/fl
Ctnnb1fl/fl CDH5CreERT2
g
Ctnnb1flex/3
x
CDH5CreERT2
Adult Ctnnb1flex/3CDH5CreERT2
P60 P61 P62 P63 P64 … P67
Tamoxifen (2mg)
h
Ctnnb1flex/3
Ctnnb1flex/3 CDH5CreERT2
100kDa
75kDa
37kDa
β–catenin
Unc5B
Netrin1
β–actin
i
β-catenin (/β-actin)
Unc5B (/β-actin)
Netrin1 (/β-actin)
Protein expression (arb.units)
p = 0.0286
NS
NS
Ctnnb1flex/3
Ctnnb1flex/3 CDH5CreERT2

Supplemental Figure 3:

(a) *Unc5B* deletion and *TCF/LEF:H2B-GFP* reporter strategy. (b) Immunofluorescence staining with the indicated antibodies and confocal imaging on P67 brain sections and quantification of ERG+ GFP levels (c). Each dot represents the mean of several images from one mouse, n=7 *Unc5B$^{fl/fl}$;TCF/LEF:H2B-GFP* and n=6 *Unc5BiECko;TCF/LEF:H2B-GFP* brains. One control mouse was set as 1. (d) *Ctnnb1* gene deletion strategy using tamoxifen injection. (e) Western blot and quantification (f) of P67 brain protein extracts, n=3 *Ctnnb1$^{fl/fl}$* and n=5 *Ctnnb1$^{fl/fl}$CDH5Cre^{ERT2}* brains. Each dot represents one mouse. One control mouse was set as 1. (g) *Ctnnb1$^{flex/3}$* overexpression strategy using tamoxifen injection. (h) Western blot and quantification (i) of P67 brain protein extracts, n=4/5 *Ctnnb1$^{flex/3}$* and n=4/5 *Ctnnb1$^{flex/3}$CDH5Cre^{ERT2}* brains. Each dot represents one mouse. One control mouse was set as 1. All data are shown as mean+/-SEM. NS: non-significant, two-sided Mann-Whitney U test was performed for statistical analysis. Source data are provided as a Source Data file.

Supp Fig. 4

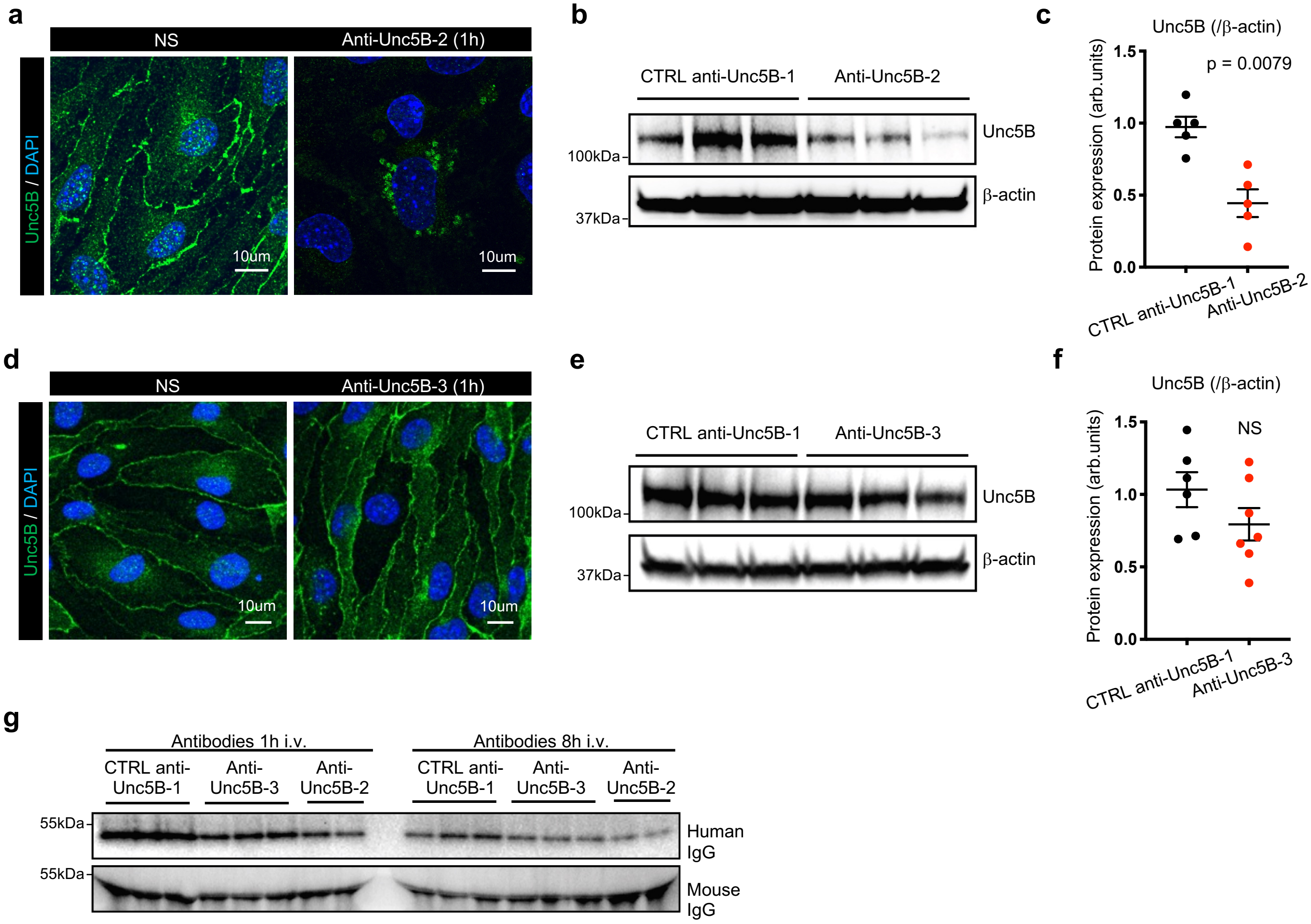
a
NS
Anti-Unc5B-2 (1h)
Unc5B / DAPI
10um
10um
b
CTRL anti-Unc5B-1
Anti-Unc5B-2
Unc5B
β-actin
100kDa
37kDa
c
Unc5B (/β-actin)
p = 0.0079
Protein expression (arb.units)
1.5
1.0
0.5
0.0
CTRL anti-Unc5B-1
Anti-Unc5B-2
d
NS
Anti-Unc5B-3 (1h)
Unc5B / DAPI
10um
10um
e
CTRL anti-Unc5B-1
Anti-Unc5B-3
Unc5B
β-actin
100kDa
37kDa
f
Unc5B (/β-actin)
NS
Protein expression (arb.units)
1.5
1.0
0.5
0.0
CTRL anti-Unc5B-1
Anti-Unc5B-3
g
Antibodies 1h i.v.
Antibodies 8h i.v.
CTRL anti-Unc5B-1
Anti-Unc5B-3
Anti-Unc5B-2
CTRL anti-Unc5B-1
Anti-Unc5B-3
Anti-Unc5B-2
55kDa
55kDa
Human IgG
Mouse IgG

Supplemental Figure 4:

(a) Unc5B immunofluorescence staining using a commercial Unc5B antibody and confocal imaging of confluent monolayers of mouse brain ECs 1h after anti-Unc5B-2 treatment, reproduced on n=4 independent experiment. (b) Western blot and quantification (c) of brain protein extracts from mice i.v injected with CTRL anti-Unc5B-1 or anti-Unc5B-2 (10 mg/kg) for 1h, n=5 CTRL anti-Unc5B-1 and n=5 anti-Unc5B-2 treated mice. Each dot represents one mouse. One control mouse was set as 1. (d) Unc5B immunofluorescence staining using a commercial Unc5B antibody and confocal imaging of confluent monolayers of mouse brain ECs 1h after anti-Unc5B-3 treatment, reproduced on n=3 independent experiment. (e) Western blot and quantification (f) of brain protein extracts from mice i.v. injected with CTRL anti-Unc5B-1 or anti-Unc5B-3 (10 mg/kg) for 1h, n=6 CTRL anti-Unc5B-1 and n=7 anti-Unc5B-3 treated mice. Each dot represents one mouse. One control mouse was set as 1. (g) Western blot of serum samples from mice i.v. injected with antibodies (10 mg/kg) for 1h. Each lane represents one mouse. All data are shown as mean+/-SEM. NS: non-significant, two-sided Mann-Whitney U test was performed for statistical analysis. Source data are provided as a Source Data file.

Supp Fig. 5

a

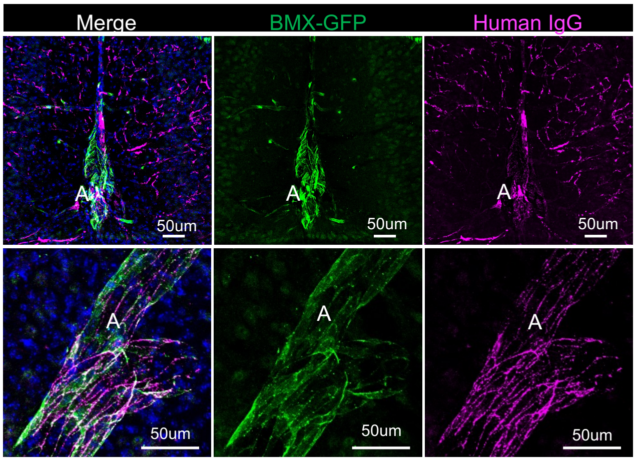

b

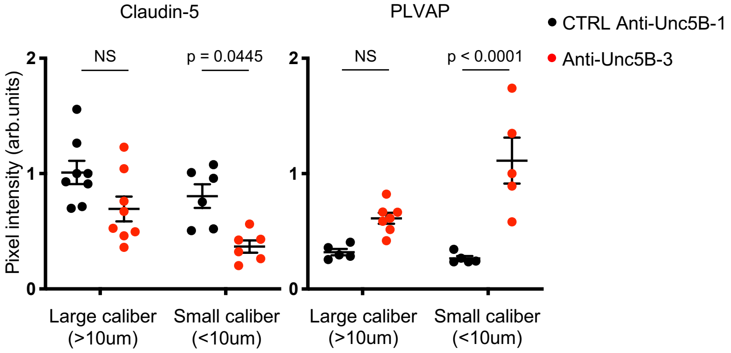

c

CTRL IgG

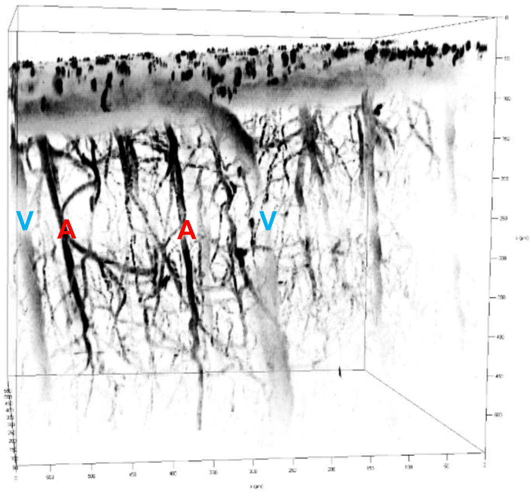

d

Anti-Unc5B-3

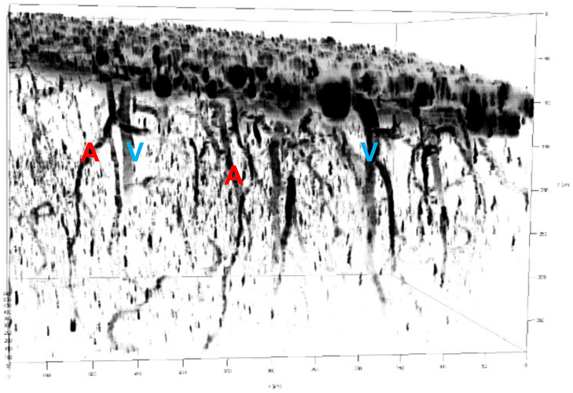

Supplemental Figure 5:

(a) Tamoxifen was injected for 5 days in *BMXCre*^{ERT2}*-mTmG* mice followed by anti-Unc5B-3 i.v. injection for 15 min. After cardiac perfusion, anti-Unc5B-3 binding was revealed by immunofluorescence using anti-human IgG antibody followed by confocal imaging and reproduced on n=3 animals. (b) Quantification of Claudin-5 and PLVAP immunostaining in larger (diameter > 10um) versus smaller vessel (diameter < 10um) on brain sections from mice i.v. injected with CTRL anti-Unc5B-1 or anti-Unc5B-3 (10 mg/kg) for 1h. Each dot represents the mean of several images, n=6/8 *Unc5B*$^{fl/fl}$ and n=6/8 *Unc5BiECko* for quantification of Claudin-5 expression and n=5 *Unc5B*$^{fl/fl}$ and n=5/7 *Unc5BiECko* for quantification of PLVAP expression (exact p-value for small caliber vessel = 0.000075). (c,d) Two-photon live imaging of WT mouse cortex 1h after i.v. injection of CTRL IgG or anti-Unc5B-3 (10 mg/kg) and 60 min after i.v. injection of 10kDa dextran. A: artery, V; Vein. NS: non-significant. All data are shown as mean+/-SEM. ANOVA followed by Bonferroni's multiple comparisons test was performed for statistical analysis between multiple groups. Source data are provided as a Source Data file.

Supp Fig. 6

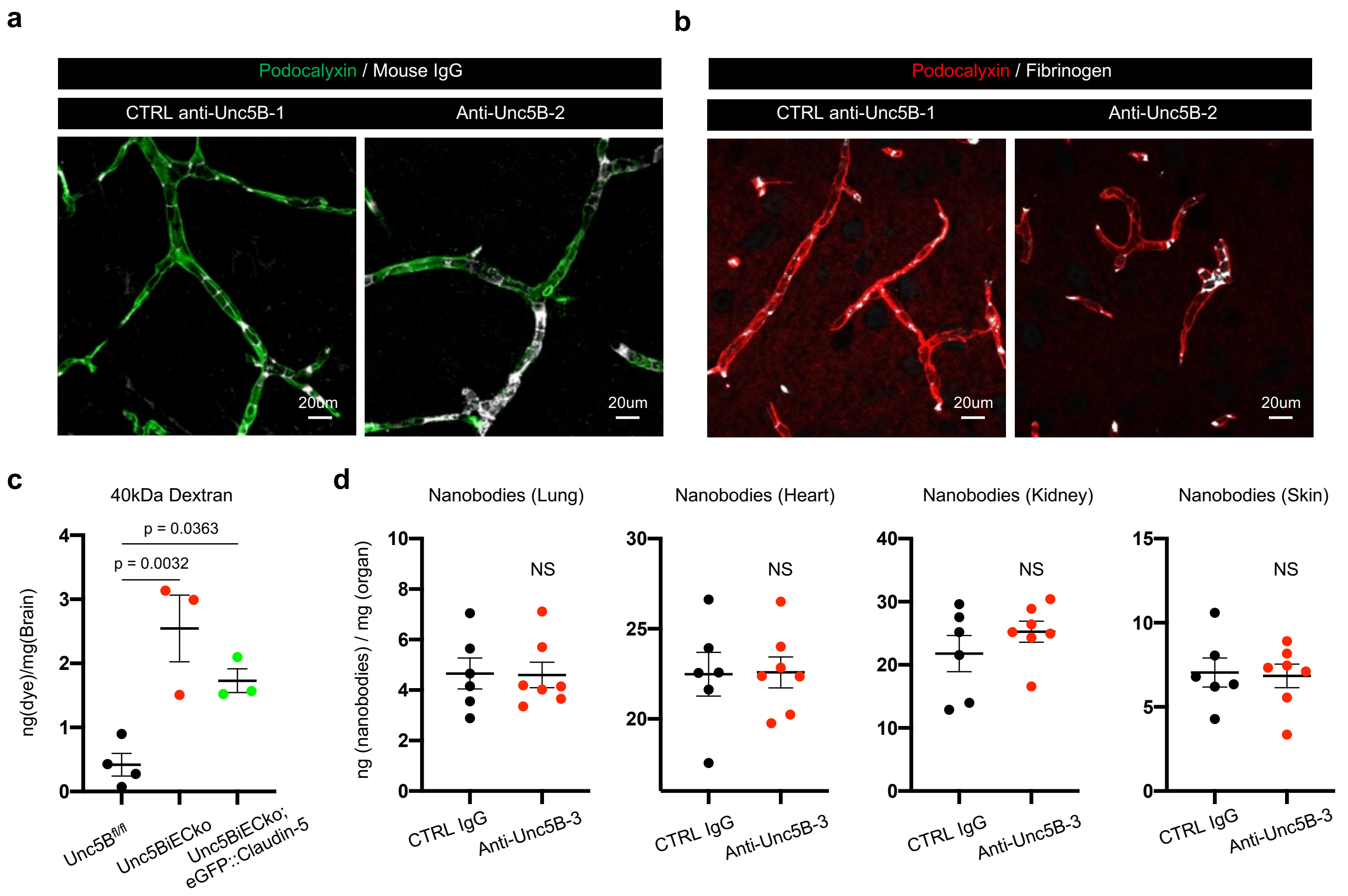

a
Podocalyxin / Mouse IgG
CTRL anti-Unc5B-1
Anti-Unc5B-2
20um
20um
b
Podocalyxin / Fibrinogen
CTRL anti-Unc5B-1
Anti-Unc5B-2
20um
20um
c
40kDa Dextran
p = 0.0363
p = 0.0032
ng(dye)/mg(Brain)
0
1
2
3
4
Unc5B fl/fl
Unc5BiECko
Unc5BiECko; eGFP::Claudin-5
d
ng (nanobodies) / mg (organ)
Nanobodies (Lung)
Nanobodies (Heart)
Nanobodies (Kidney)
Nanobodies (Skin)
NS
NS
NS
NS
0
2
4
6
8
10
20
25
30
0
10
20
30
40
0
5
10
15
CTRL IgG
Anti-Unc5B-3

Supplemental Figure 6:

(a,b) Immunofluorescence staining with the indicated antibodies and confocal imaging on brain sections from mice i.v. injected with CTRL anti-Unc5B-1 or anti-Unc5B-2 for 1h, reproduced on n=4 CTRL anti-Unc5B-1 and n=5 anti-Unc5B-2 treated animals. (c) Quantification of 40kDa dextran content in P67 brains, 30 min after i.v. 40kDa dextran injection, n=4 *Unc5B*$^{fl/fl}$, n=3 *Unc5BiECko* and n=3 *Unc5BiECko;eGFP::Claudin-5* brains. Each dot represents one mouse. (d) Quantification of nanobody content in peripheral organs, 1h after i.v. CTRL IgG or anti-Unc5B-3 injection (10 mg/kg) and 30 min after i.v. nanobody injection, n=6 CTRL IgG and n=7 anti-Unc5B-3 treated mice. Each dot represents one mouse. All data are shown as mean+/-SEM. NS: non-significant, two-sided Mann-Whitney U test was performed for statistical analysis. ANOVA followed by Bonferroni's multiple comparisons test was performed for statistical analysis between multiple groups. Source data are provided as a Source Data file.

Supp Fig. 7

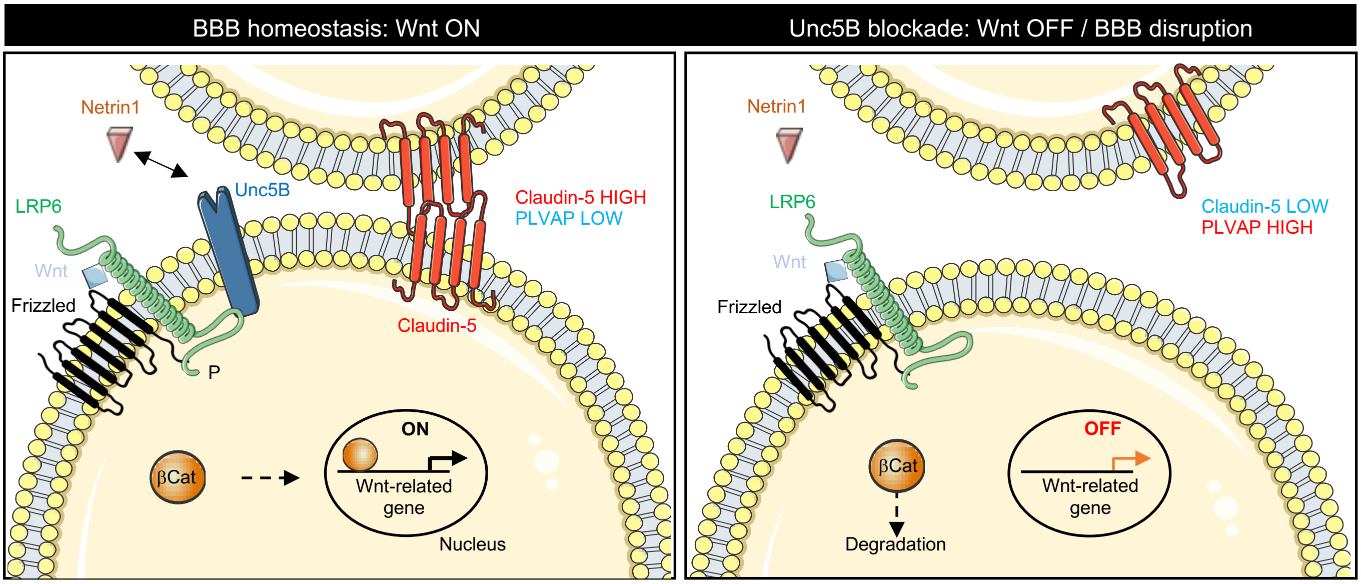

BBB homeostasis: Wnt ON
Unc5B blockade: Wnt OFF / BBB disruption
Netrin1
Unc5B
LRP6
Wnt
Frizzled
P
Claudin-5 HIGH
PLVAP LOW
Claudin-5
ON
βCat
Wnt-related
gene
Nucleus
Netrin1
LRP6
Wnt
Frizzled
Claudin-5 LOW
PLVAP HIGH
βCat
Degradation
OFF
Wnt-related
gene

Supplemental Figure 7:

Working model. Netrin-1 binding to endothelial Unc5B maintains BBB integrity, by promoting LRP6 phosphorylation and Wnt/β-catenin-induced expression of Claudin-5 and repression of PLVAP. In the absence of Netrin-1-Unc5B signaling, the Wnt/β-catenin signaling is disrupted which induced loss of Claudin-5 along with increased PLVAP expression and BBB leakage. Figure was created using Servier Medical Art (smart.servier.com), licensed under a Creative Commons Attribution 3.0 Unported License

Supp Fig. 8

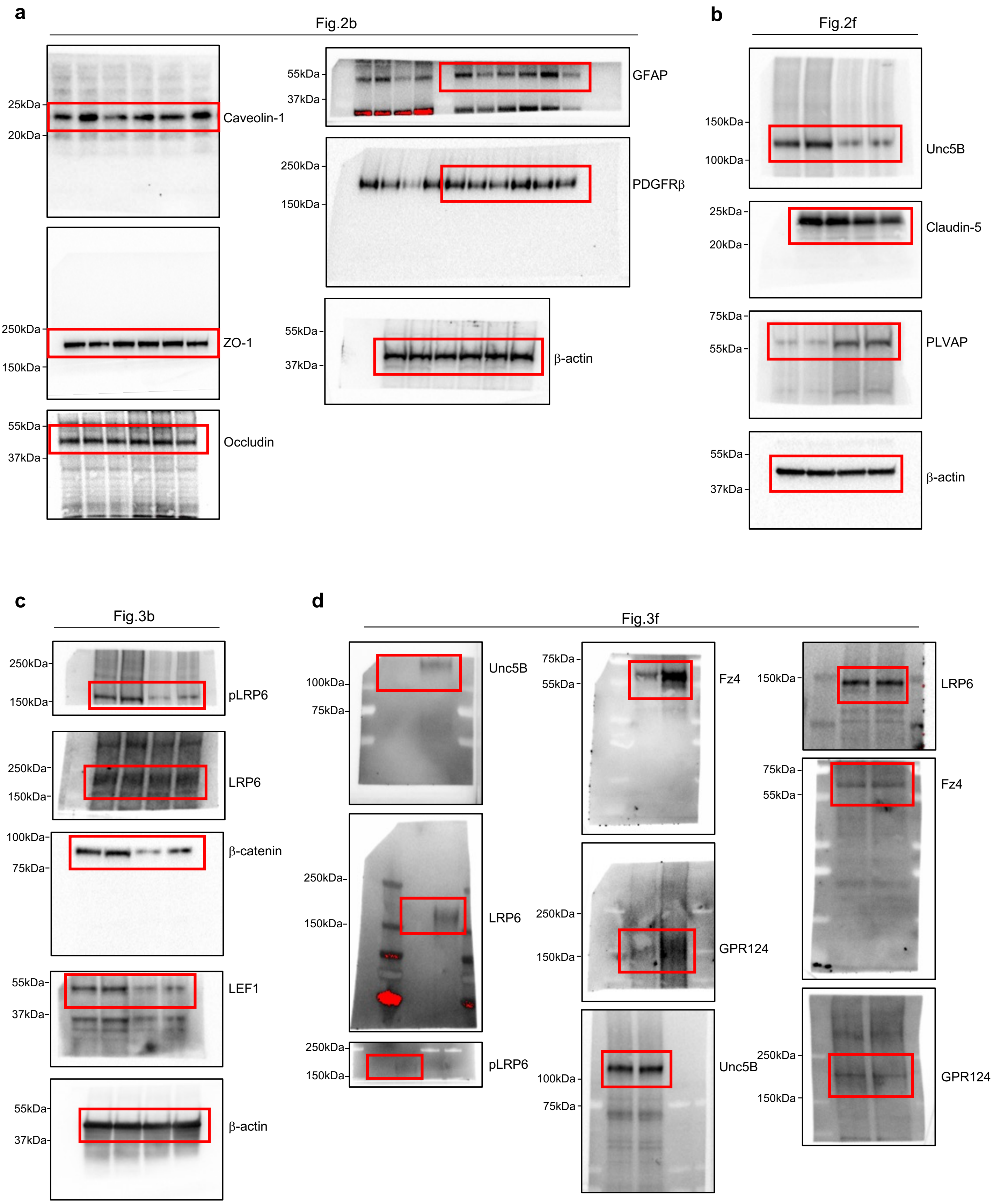
a
Fig.2b
25kDa
20kDa
Caveolin-1
250kDa
150kDa
ZO-1
55kDa
37kDa
Occludin
55kDa
37kDa
GFAP
250kDa
150kDa
PDGFRβ
55kDa
37kDa
β-actin
b
Fig.2f
150kDa
100kDa
Unc5B
25kDa
20kDa
Claudin-5
75kDa
55kDa
PLVAP
55kDa
37kDa
β-actin
c
Fig.3b
250kDa
150kDa
pLRP6
250kDa
150kDa
LRP6
100kDa
75kDa
β-catenin
55kDa
37kDa
LEF1
55kDa
37kDa
β-actin
d
Fig.3f
100kDa
75kDa
Unc5B
250kDa
150kDa
LRP6
250kDa
150kDa
pLRP6
75kDa
55kDa
Fz4
250kDa
150kDa
GPR124
100kDa
75kDa
Unc5B
150kDa
LRP6
75kDa
55kDa
Fz4
250kDa
150kDa
GPR124

Supplemental Figure 8:

Uncropped western blots from (a) Fig. 2b, (b) Fig. 2f, (c) Fig. 3b, (d) Fig. 3f

Supp Fig. 9

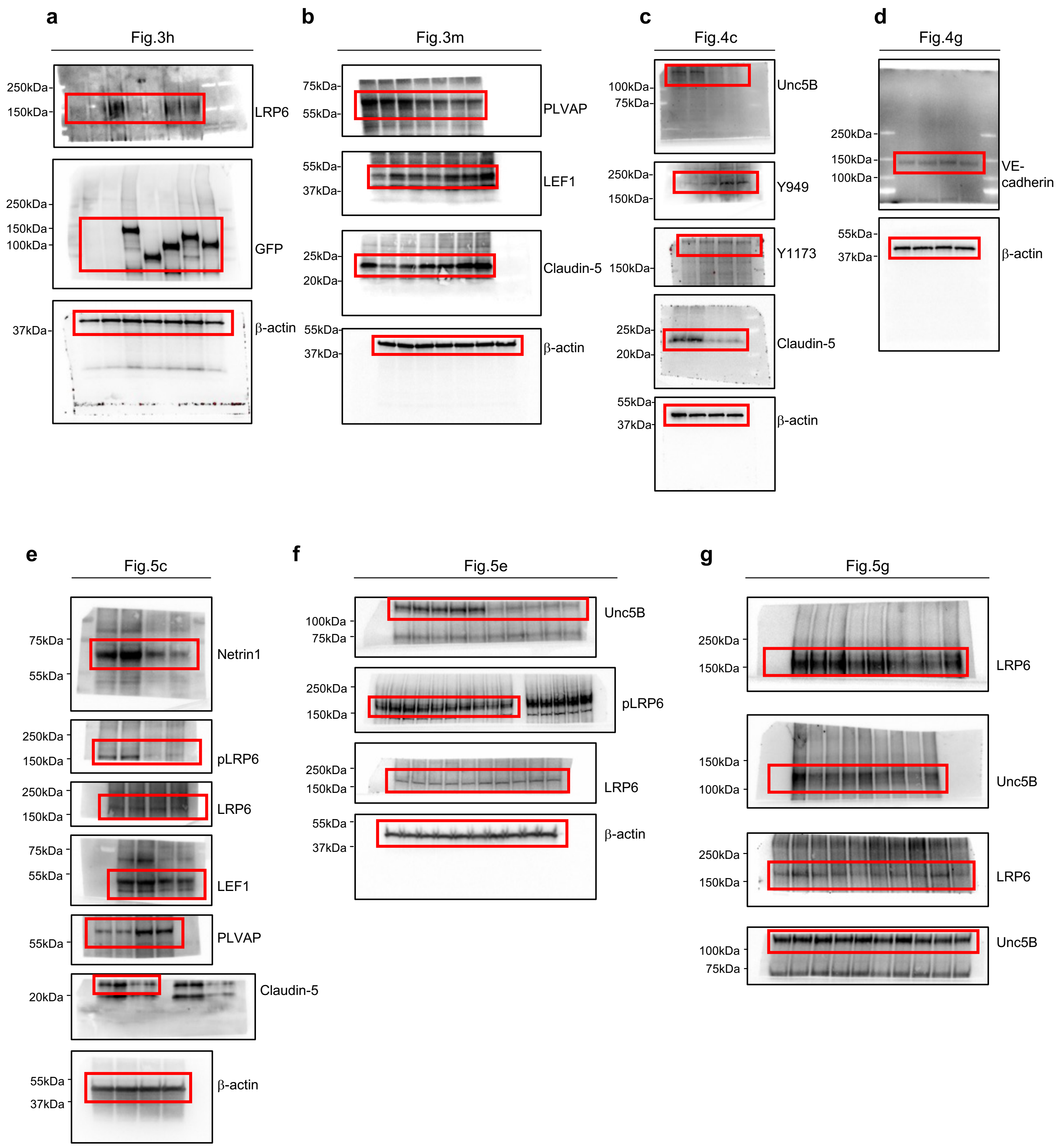

a
Fig.3h
250kDa
150kDa
LRP6
250kDa
150kDa
100kDa
GFP
37kDa
β-actin
b
Fig.3m
75kDa
55kDa
PLVAP
55kDa
37kDa
LEF1
25kDa
20kDa
Claudin-5
55kDa
37kDa
β-actin
c
Fig.4c
100kDa
75kDa
Unc5B
250kDa
150kDa
Y949
150kDa
Y1173
25kDa
20kDa
Claudin-5
55kDa
37kDa
β-actin
d
Fig.4g
250kDa
150kDa
100kDa
VE-cadherin
55kDa
37kDa
β-actin
e
Fig.5c
75kDa
55kDa
Netrin1
250kDa
150kDa
pLRP6
250kDa
150kDa
LRP6
75kDa
55kDa
LEF1
55kDa
PLVAP
20kDa
Claudin-5
55kDa
37kDa
β-actin
f
Fig.5e
100kDa
75kDa
Unc5B
250kDa
150kDa
pLRP6
250kDa
150kDa
LRP6
55kDa
37kDa
β-actin
g
Fig.5g
250kDa
150kDa
LRP6
150kDa
100kDa
Unc5B
250kDa
150kDa
LRP6
100kDa
75kDa
Unc5B

Supplemental Figure 9:

Uncropped western blots from (a) Fig. 3h, (b) Fig. 3m, (c) Fig. 4c, (d) Fig. 4g, (e) Fig. 5c, (f) Fig. 5e, (g) Fig. 5g

Supp Fig. 10

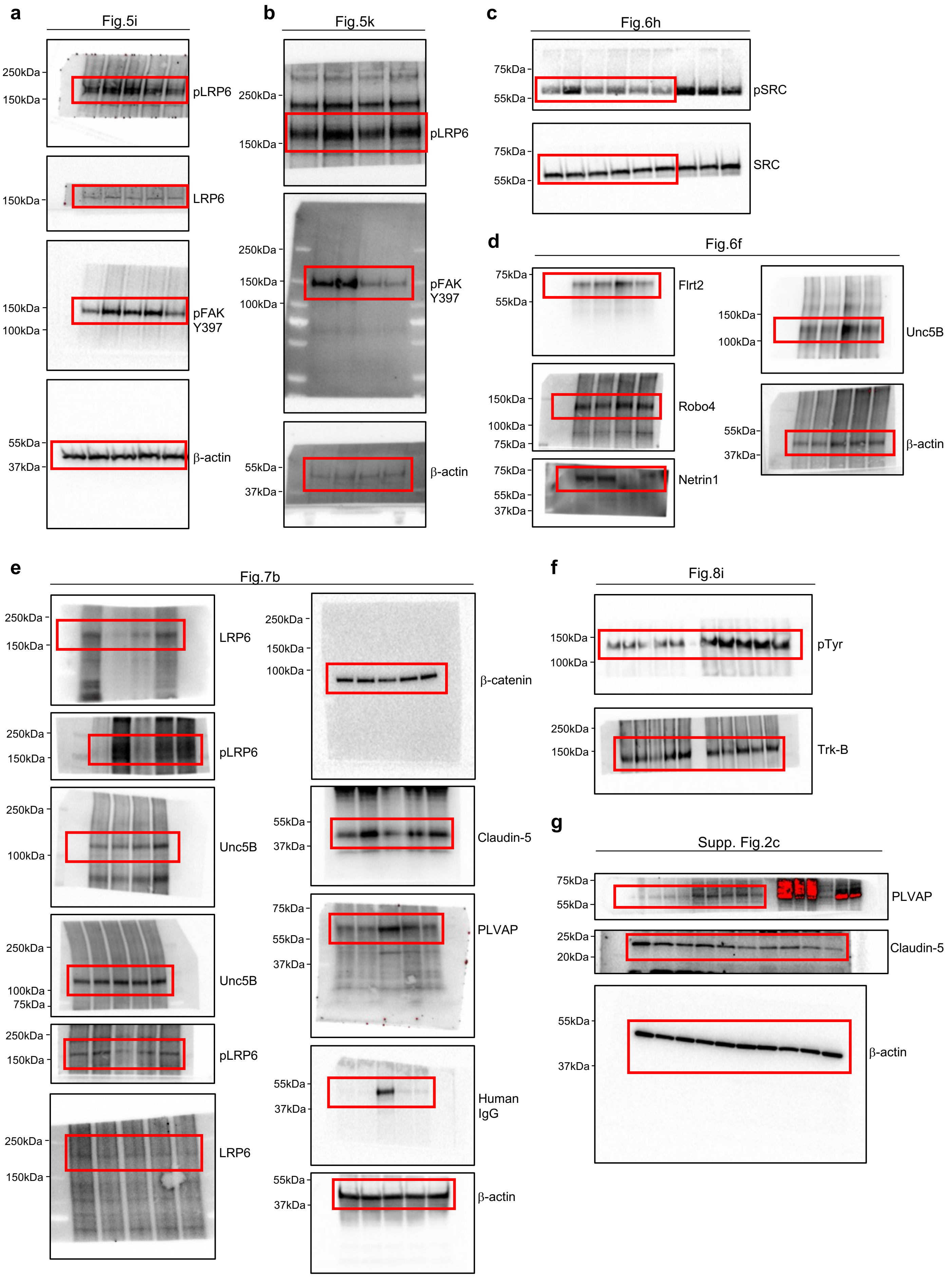
a
Fig.5i
250kDa
150kDa
pLRP6
150kDa
LRP6
150kDa
100kDa
pFAK Y397
55kDa
37kDa
β-actin
b
Fig.5k
250kDa
150kDa
pLRP6
250kDa
150kDa
100kDa
pFAK Y397
55kDa
37kDa
β-actin
c
Fig.6h
75kDa
55kDa
pSRC
75kDa
55kDa
SRC
d
Fig.6f
75kDa
55kDa
Flrt2
150kDa
100kDa
75kDa
Robo4
75kDa
55kDa
37kDa
Netrin1
150kDa
100kDa
Unc5B
55kDa
37kDa
β-actin
e
Fig.7b
250kDa
150kDa
LRP6
250kDa
150kDa
pLRP6
250kDa
100kDa
Unc5B
250kDa
100kDa
75kDa
Unc5B
250kDa
150kDa
pLRP6
250kDa
150kDa
LRP6
250kDa
150kDa
100kDa
β-catenin
55kDa
37kDa
Claudin-5
75kDa
55kDa
37kDa
PLVAP
55kDa
37kDa
Human IgG
55kDa
37kDa
β-actin
f
Fig.8i
150kDa
100kDa
pTyr
250kDa
150kDa
Trk-B
g
Supp. Fig.2c
75kDa
55kDa
PLVAP
25kDa
20kDa
Claudin-5
55kDa
37kDa
β-actin

Supplemental Figure 10:

Uncropped western blots from (a) Fig. 5i, (b) Fig. 5k, (c) Fig. 6h, (d) Fig. 6f, (e) Fig. 7b, (f) Fig. 8i, (g) Supp. Fig. 2c

Supp Fig.11

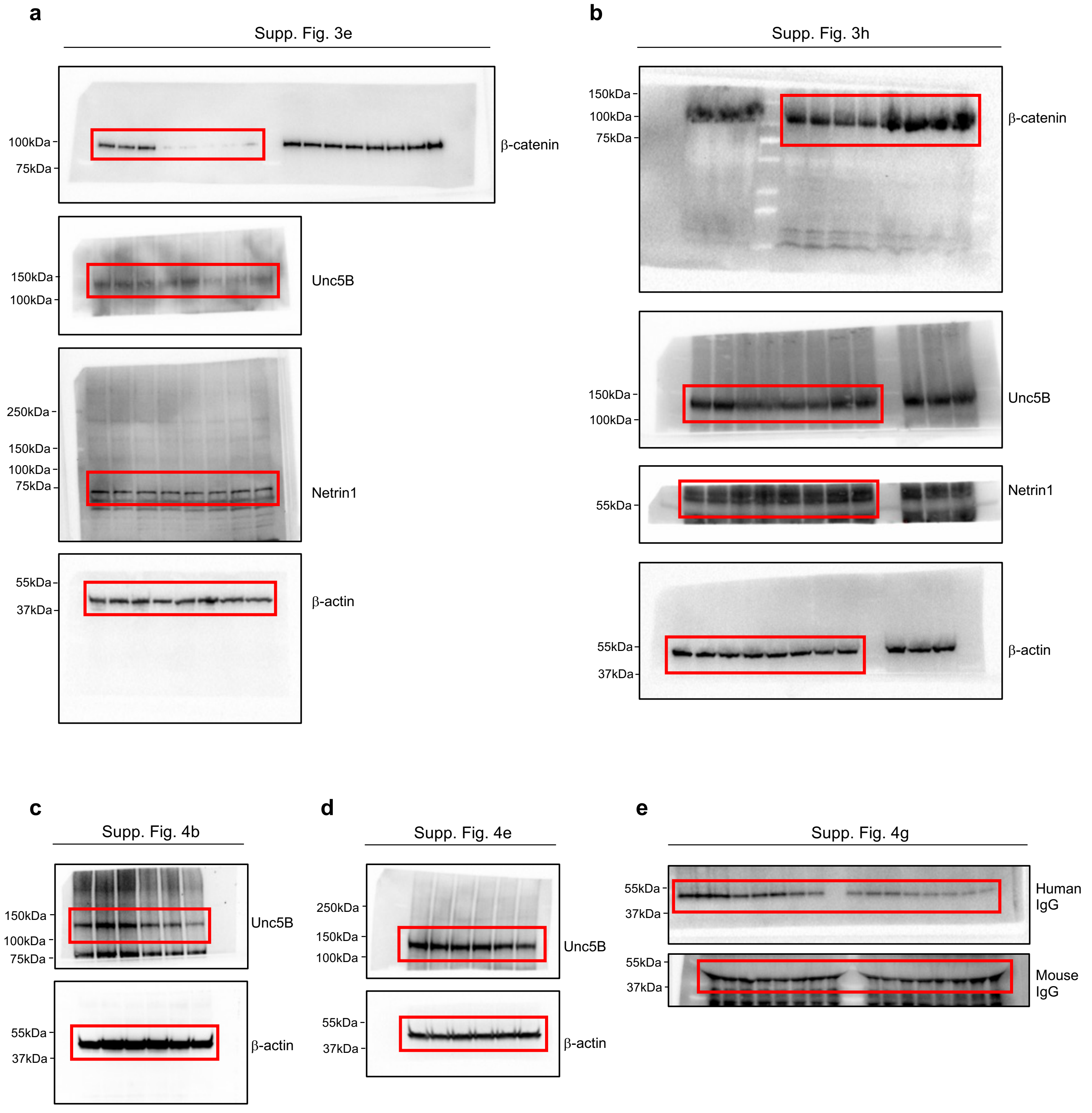

a
Supp. Fig. 3e
100kDa
75kDa
β-catenin
150kDa
100kDa
Unc5B
250kDa
150kDa
100kDa
75kDa
Netrin1
55kDa
37kDa
β-actin
b
Supp. Fig. 3h
150kDa
100kDa
75kDa
β-catenin
150kDa
100kDa
Unc5B
55kDa
Netrin1
55kDa
37kDa
β-actin
c
Supp. Fig. 4b
150kDa
100kDa
75kDa
Unc5B
55kDa
37kDa
β-actin
d
Supp. Fig. 4e
250kDa
150kDa
100kDa
Unc5B
55kDa
37kDa
β-actin
e
Supp. Fig. 4g
55kDa
37kDa
Human IgG
55kDa
37kDa
Mouse IgG

Supplemental Figure 11:

Uncropped western blots from (a) Supp. Fig. 3e, (b) Supp. Fig. 3h, (c) Supp. Fig. 4b, (d) Supp. Fig. 4e, (e) Supp. Fig. 4g
