# Supplementary material for: Endothelial Unc5B controls blood-brain barrier integrity
Source: Nat Commun. 2022 Mar 4;13:1169. doi: 10.1038/s41467-022-28785-9 (PMC8897508; doi:10.1038/s41467-022-28785-9)
Supplement: Supplementary file 3 — Description of Additional Supplementary Files [file 41467_2022_28785_MOESM3_ESM.docx]

**Description of Additional Supplementary Files:**

**Supplementary Movie:** Unc5B gene deletion was induced by tamoxifen injection from P0-2 and P10 Unc5BiECko neonates were videorecorded. Please note that Unc5BiECko neonates exhibit unresponsive freezing, loss of balance and consciousness, reproduced on a total of n=7 Unc5BiECko neonates.
