# Supplementary material for: Endothelial Unc5B controls blood-brain barrier integrity
Source: Nat Commun. 2022 Mar 4;13:1169. doi: 10.1038/s41467-022-28785-9 (PMC8897508; doi:10.1038/s41467-022-28785-9)
Supplement: Supplementary file 5 — Reporting Summary [file 41467_2022_28785_MOESM5_ESM.pdf]

nature research

Corresponding author(s): Anne Eichmann

Last updated by author(s): Nov 23, 2021

# Reporting Summary

Nature Research wishes to improve the reproducibility of the work that we publish. This form provides structure for consistency and transparency in reporting. For further information on Nature Research policies, see our Editorial Policies and the Editorial Policy Checklist.

## Statistics

For all statistical analyses, confirm that the following items are present in the figure legend, table legend, main text, or Methods section.

| n/a | Confirmed | |
|---|---|---|
| [ ] | [x] | The exact sample size ($n$) for each experimental group/condition, given as a discrete number and unit of measurement |
| [ ] | [x] | A statement on whether measurements were taken from distinct samples or whether the same sample was measured repeatedly |
| [ ] | [x] | The statistical test(s) used AND whether they are one- or two-sided<br>*Only common tests should be described solely by name; describe more complex techniques in the Methods section.* |
| [ ] | [x] | A description of all covariates tested |
| [ ] | [x] | A description of any assumptions or corrections, such as tests of normality and adjustment for multiple comparisons |
| [ ] | [x] | A full description of the statistical parameters including central tendency (e.g. means) or other basic estimates (e.g. regression coefficient) AND variation (e.g. standard deviation) or associated estimates of uncertainty (e.g. confidence intervals) |
| [ ] | [x] | For null hypothesis testing, the test statistic (e.g. $F$, $t$, $r$) with confidence intervals, effect sizes, degrees of freedom and $P$ value noted<br>*Give P values as exact values whenever suitable.* |
| [x] | [ ] | For Bayesian analysis, information on the choice of priors and Markov chain Monte Carlo settings |
| [x] | [ ] | For hierarchical and complex designs, identification of the appropriate level for tests and full reporting of outcomes |
| [x] | [ ] | Estimates of effect sizes (e.g. Cohen's $d$, Pearson's $r$), indicating how they were calculated |

*Our web collection on statistics for biologists contains articles on many of the points above.*

## Software and code

Policy information about availability of computer code

| | |
|---|---|
| Data collection | All data were collected using the following device or software:<br>- Western blot bands were acquired on a Biorad Gel Doc EQ System with Universal Hood II imaging system equipped with Image Lab v5.2.1 software.<br>- Binding of anti-Unc5B antibodies to Human or Rat Unc5B was performed using a BiacoreTM 8K (Proteogenix, Schiltigheim, France)<br>- Dye extravasations were performed either at the Yale Cardiovascular Research Center (New Haven, CT, USA) on a BioTek synergy2 spectrophotometer or at the Paris Cardiovascular Research Center (Paris, France) on a Flexstation3 spectrophotometer<br>- Confocal images were acquired on a laser scanning fluorescence microscope (Leica SP8 and Zeiss LSM800) using the appropriate software (LAS-X or ZEN 2 blue edition system)<br>- Two-photon live imaging was performed using a Leica SP8 DIVE in vivo imaging system equipped with 4tune spectral external hybrid detectors and an InSightX3 laser (SpectraPhysics)<br>- Vascular density data was collected with the software Angiotool v0.5, vessel diameter data was collected on Leica LAS-X software, all other immunostaining data collections were performed using the software ImageJ 1.52q. |
| Data analysis | All graphs and statistical analysis were done using GraphPad Prism 8 software |

For manuscripts utilizing custom algorithms or software that are central to the research but not yet described in published literature, software must be made available to editors and reviewers. We strongly encourage code deposition in a community repository (e.g. GitHub). See the Nature Research guidelines for submitting code & software for further information.

## Data

Policy information about availability of data

All manuscripts must include a data availability statement. This statement should provide the following information, where applicable:

- Accession codes, unique identifiers, or web links for publicly available datasets
- A list of figures that have associated raw data
- A description of any restrictions on data availability

All data generated are included in this article (main or supplementary information files) and are provided in the Source Data file. Additional information can be obtained from the corresponding author.

# Field-specific reporting

Please select the one below that is the best fit for your research. If you are not sure, read the appropriate sections before making your selection.

☒ Life sciences ☐ Behavioural & social sciences ☐ Ecological, evolutionary & environmental sciences

For a reference copy of the document with all sections, see nature.com/documents/nr-reporting-summary-flat.pdf

# Life sciences study design

All studies must disclose on these points even when the disclosure is negative.

| | |
|---|---|
| Sample size | We did not used any statistical methods to predetermine sample size. The determination of sample size is based on our experience and known literature, to reach an appropriate "n" numbers of time allowing us to perform statistical analysis.<br>All in vitro experiment were reproduced and quantified on n=4 independent experiment (n=3 for Figure 5k,i)<br>All in vivo experiment were reproduced and quantified on a minimum or n=3/4 mice per conditions (n=5 mice or more for most of the experiments) |
| Data exclusions | No data were excluded in this manuscripts |
| Replication | A minimum of n=3/4 independent experiment were performed for in vitro experiment, while a minimum of n=3/4 animal were used for in vivo studies. Furthermore, all main experimental finding were reproduced by another laboratory (Paris Cardiovascular Research Center) which include (but is not limited to) :<br>- Blood-Brain Barrier leakage assay for Cadaverine, 10- 40 and 70-kDa dextran extravasation<br>- Western blot from brain protein extracts showing decreased wnt signaling upon Unc5B blockade<br>- Unc5B/LRP6 immunoprecipitation from brain endothelial cells<br>All replication were successful. |
| Randomization | Mice with genetic modifications were not randomized and were allocated to their respective groups based on their genotype. For antibody-treated mice, mice were randomly allocated to experimental groups prior to antibody treatement. |
| Blinding | Investigators were not blinded to group allocations for data collection as they needed to inject specific drugs or to use specific genetically modified mice. However, investigators were blinded for data analysis of immunofluorescence staining and quantification of dye extravasations and all main experiments were successfully reproduced by independent investigators from another laboratory (Paris Cardiovascular Research Center). |

# Reporting for specific materials, systems and methods

We require information from authors about some types of materials, experimental systems and methods used in many studies. Here, indicate whether each material, system or method listed is relevant to your study. If you are not sure if a list item applies to your research, read the appropriate section before selecting a response.

## Materials & experimental systems

| n/a | Involved in the study | |
|---|---|---|
| ☐ | ☒ | Antibodies |
| ☐ | ☒ | Eukaryotic cell lines |
| ☒ | ☐ | Palaeontology and archaeology |
| ☐ | ☒ | Animals and other organisms |
| ☒ | ☐ | Human research participants |
| ☒ | ☐ | Clinical data |
| ☒ | ☐ | Dual use research of concern |

## Methods

| n/a | Involved in the study | |
|---|---|---|
| ☒ | ☐ | ChIP-seq |
| ☒ | ☐ | Flow cytometry |
| ☒ | ☐ | MRI-based neuroimaging |

## Antibodies

Antibodies used

The following antibodies were incubated overnight at 4°C for western blots and immunoprecipitations: Unc5B (Cell Signaling, 13851S, dilution: 1/1000), Unc5B (R&D, AF1006, dilution: 1/200), Robo4 (Invitrogen, 20221-1-AP, dilution: 1/300), Flrt2 (Novus bio, NBP2-43653, dilution: 1/500), Netrin-1 (R&D, AF1109, dilution: 1/500), Claudin-5 (Invitrogen, 35-2500, dilution: 1/1000), PLVAP (BD biosciences, 550563, dilution: 1/200), PDGFRb (Cell Signaling, 3169S, dilution: 1/1000), GFAP (Millipore, MAB360, dilution: 1/1000), VEGFR2 Y949 (Cell Signaling, 4991S, dilution: 1/500), VEGFR2 Y1173 (Cell Signaling, 2478S, dilution: 1/500), pLRP6 (Cell Signaling, 2568S, dilution: 1/300), LRP6 (Cell Signaling, 3395S, dilution: 1/500), b-catenin (Cell Signaling, 8480S, dilution: 1/2000), LEF1 (Cell Signaling, 2230S, dilution: 1/1000), ZO1 (Invitrogen, 61-7300, dilution: 1/2000), Occludin (Invitrogen, 33-1500, dilution: 1/1000), Caveolin-1 (Cell Signaling, 3238S, dilution: 1/2000), VE-cadherin (BD Pharmingen, 555289, dilution: 1/500) and bactin (Sigma, A1978, dilution: 1/5000). Then, membranes were washed 4 x 10min in TBS 0.1% Tween and incubated with one of the following peroxidase-conjugated secondary antibodies diluted 1/5000 in TBS 0.1% Tween supplemented with 5%BSA for 2h at room temperature: horse anti-mouse IgG(H+L) (Vector laboratories, PI-2000), goat anti-rabbit IgG(H+L) (Vector laboratories, PI-1000), goat anti-rat IgG(H+L) (Vector laboratories, PI-9400), horse anti-goat IgG(H+L) (Vector laboratories, PI-9500).

The following antibodies were used for immunostaining: Podocalyxin (RD, AF1556, dilution: 1/400), Unc5B (Cell signaling, 13851S, dilution: 1/200), Claudin-5-GFP (Invitrogen, 352588, dilution: 1/200), PLVAP (BD biosciences, 550563, dilution: 1/100), LEF1 (Cell Signaling, 2230S, dilution: 1/200), GFAP (Millipore, MAB360, dilution: 1/400), Aquaporin-4 (Millipore, AB3068, dilution: 1/400), PDGFRb (Cell Signaling, 3169S, dilution: 1/400), Endomucin (Hycult biotech, HM1108, dilution: 1/400), fibrinogen (DAKO, A0080, dilution: 1/400), CD13 (BD Biosciences, 558744, dilution: 1/200), VE-cadherin (BD Pharmingen, 555289, dilution: 1/200), DAPI (Thermo Fischer, 62248, dilution: 1/2000). All corresponding secondary antibodies were purchased from Invitrogen as donkey anti-primary antibody coupled with either Alexa Fluor 488, 568 or 647 and were diluted 1/200.

Validation

Antibodies used in this studies were all reported and validated by the manufacturer and in the literature
-Unc5B (Cell Signaling, 13851S) https://www.cellsignal.com/products/primary-antibodies/unc5b-d9m7z-rabbit-mab/13851
-Unc5B (R&D, AF1006) https://www.rndsystems.com/products/rat-unc5h2-unc5b-antibody_af1006
-Robo4 (Invitrogen, 20221-1-AP) https://www.thermofisher.com/antibody/product/ROBO4-Antibody-Polyclonal/20221-1-AP
-Flrt2 (Novus bio, NBP2-43653) https://www.novusbio.com/products/flrt2-antibody_nbp2-43653
-Netrin-1 (R&D, AF1109) https://www.rndsystems.com/products/mouse-netrin-1-antibody_af1109
-Claudin-5 (Invitrogen, 35-2500) https://www.thermofisher.com/order/genome-database/details/antibody/35-2500.html
-PLVAP (BD biosciences, 550563) https://www.bdbiosciences.com/en-us/products/reagents/flow-cytometry-reagents/research-reagents/single-color-antibodies-ruo/purified-rat-anti-mouse-panendothelial-cell-antigen.550563
-PDGFRb (Cell Signaling, 3169S) https://www.cellsignal.com/products/primary-antibodies/pdgf-receptor-b-28e1-rabbit-mab/3169
-GFAP (Millipore, MAB360) https://www.emdmillipore.com/US/en/product/Anti-Glial-Fibrillary-Acidic-Protein-Antibody-clone-GA5,MM_NF-MAB360?ReferrerURL=https%3A%2F%2Fwww.google.com%2F&bd=1
-VEGFR2 Y949 (Cell Signaling, 4991S) https://www.cellsignal.com/products/primary-antibodies/phospho-vegf-receptor-2-tyr951-15d2-rabbit-mab/4991
-VEGFR2 Y1173 (Cell Signaling, 2478S) https://www.cellsignal.com/products/primary-antibodies/phospho-vegf-receptor-2-tyr1175-19a10-rabbit-mab/2478
-pLRP6 (Cell Signaling, 2568S) https://www.cellsignal.com/products/primary-antibodies/phospho-lrp6-ser1490-antibody/2568
-LRP6 (Cell Signaling, 3395S) https://www.cellsignal.com/products/primary-antibodies/lrp6-c47e12-rabbit-mab/3395
-b-catenin (Cell Signaling, 8480S) https://www.cellsignal.com/products/primary-antibodies/b-catenin-d10a8-xp-rabbit-mab/8480
-LEF1 (Cell Signaling, 2230S) https://www.cellsignal.com/products/primary-antibodies/lef1-c12a5-rabbit-mab/2230
-ZO1 (Invitrogen, 61-7300) https://www.thermofisher.com/antibody/product/ZO-1-Antibody-Polyclonal/61-7300
-Occludin (Invitrogen, 33-1500) https://www.thermofisher.com/antibody/product/Occludin-Antibody-clone-OC-3F10-Monoclonal/33-1500
-Caveolin-1 (Cell Signaling, 3238S) https://www.cellsignal.com/products/primary-antibodies/caveolin-1-antibody/3238
- VE-cadherin (BD Pharmingen, 555289) https://www.bdbiosciences.com/en-us/products/reagents/functional-cell-based-reagents/purified-rat-anti-mouse-cd144.555289
-bactin (Sigma, A1978) https://www.sigmaaldrich.com/US/en/product/sigma/a1978
-horse anti-mouse IgG(H+L) (Vector laboratories, PI-2000) https://vectorlabs.com/peroxidase-horse-anti-mouse-igg-antibody.html
-goat anti-rabbit IgG(H+L) (Vector laboratories, PI-1000) https://vectorlabs.com/peroxidase-goat-anti-rabbit-igg-antibody.html
-goat anti-rat IgG(H+L) (Vector laboratories, PI-9400) https://vectorlabs.com/peroxidase-goat-anti-rat-igg-antibody.html
-horse anti-goat IgG(H+L) (Vector laboratories, PI-9500) https://vectorlabs.com/peroxidase-horse-anti-goat-igg-antibody.html
-Podocalyxin (RD, AF1556) https://www.rndsystems.com/products/mouse-podocalyxin-antibody_af1556
-Claudin-5-GFP (Invitrogen, 352588) https://www.thermofisher.com/antibody/product/Claudin-5-Antibody-clone-4C3C2-Monoclonal/352588
-Aquaporin-4 (Millipore, AB3068, dilution: 1/400) https://www.emdmillipore.com/US/en/product/Aquaporin-4-control-peptide-for-AB3068,MM_NF-AG777?ReferrerURL=https%3A%2F%2Fwww.google.com%2F&bd=1
-Endomucin (Hycult biotech, HM1108) https://www.hycultbiotech.com/hm1108-100ug
-Fibrinogen (DAKO, A0080) https://www.agilent.com/en/product/specific-proteins/multipurpose-antibodies-for-clinical-chemistry/fibrinogen-(multipurpose)-76975
-CD13 (BD Biosciences, 558744) https://www.bdbiosciences.com/en-us/products/reagents/flow-cytometry-reagents/research-reagents/single-color-antibodies-ruo/fitc-rat-anti-mouse-cd13.558744
- DAPI (Thermo Fischer, 62248) https://www.thermofisher.com/order/catalog/product/62248

# Eukaryotic cell lines

Policy information about cell lines

| | |
|---|---|
| Cell line source(s) | C57BL/6 Mouse Primary Brain Microvascular Endothelial Cells were purchased from Cell Biologics (C57-6023) |
| Authentication | C57BL/6 Mouse Primary Brain Microvascular Endothelial Cells were authemticated by Cell Biologics before use.<br>We also validated expression of gene enriched in the brain endothelium compared to traditional HUVEC cells.<br>Furthermore, this cell line is commonly used and is a validated cell line for Blood-Brain Barrier in vitro studies. |
| Mycoplasma contamination | Cells were routinely check for mycoplama contamination. No experiment were performed on mycoplasma positive cells. |
| Commonly misidentified lines<br>(See ICLAC register) | No commonly misidentified lines were used. |

# Animals and other organisms

Policy information about studies involving animals; ARRIVE guidelines recommended for reporting animal research

| | |
|---|---|
| Laboratory animals | The following mice were used for this studies:<br>- Unc5Bfl/fl-Cdh5CreERT2 mice<br>- Unc5Bfl/fl-PdgfrbCreERT2 mice<br>- Robo4+/- mice<br>- Netrin1fl/fl-RosaCreERT2 mice<br>- Unc5Bfl/fl-Cdh5CreERT2;eGFP::Claudin5 mice<br>- Unc5Bfl/fl-Cdh5CreERT2;Y949F mice<br>- Unc5Bfl/fl-Cdh5CreERT2;Ctnnb1flex/3 mice<br>- Unc5Bfl/fl-Cdh5CreERT2;Ctnnb1fl/fl mice<br>- Ctnnb1flex/3;Cdh5CreERT2 mice<br>- Ctnnb1fl/fl;Cdh5CreERT2 mice<br>- Wild type mice<br>Animals were housed at 20–24 °C, with 30–70% humidity under a 12h light-dark cycle. Animal from both sexes were used for this studies.<br>For postnatal experiment, animal were used at postnatal day (P)5 or 12. For adult experiment, 8 to 10 weeks old mice were used. |
| Wild animals | No wild animals were used in the study. |
| Field-collected samples | No field collected samples were used in the study. |
| Ethics oversight | All protocols and experimental procedures were approved by the Yale University Institutional Animal Care and Use Committee (IACUC). |

Note that full information on the approval of the study protocol must also be provided in the manuscript.
